# Attenuated heartbeat-evoked potentials in functional neurological disorder

**DOI:** 10.1093/braincomms/fcaf503

**Published:** 2026-01-03

**Authors:** Natascha Stoffel, Michaël Mouthon, Hang Yang, Laure von der Weid, Cristina Concetti, Olaf Blanke, Selma Aybek

**Affiliations:** Faculty of Science and Medicine, Department of Neurology, University of Fribourg, Fribourg 1700, Switzerland; Graduate School for Health Sciences (GHS), University of Bern, Bern 3012, Switzerland; Faculty of Science and Medicine, Department of Neurology, University of Fribourg, Fribourg 1700, Switzerland; Laboratory of Cognitive Neuroscience, Brain Mind Institute, École Polytechnique Fédérale de Lausanne (EPFL), Geneva 1202, Switzerland; Faculty of Science and Medicine, Department of Neurology, University of Fribourg, Fribourg 1700, Switzerland; Faculty of Science and Medicine, Department of Neurology, University of Fribourg, Fribourg 1700, Switzerland; Laboratory of Cognitive Neuroscience, Brain Mind Institute, École Polytechnique Fédérale de Lausanne (EPFL), Geneva 1202, Switzerland; Department of Clinical Neurosciences, University Hospital of Geneva, Geneva 1211, Switzerland; Faculty of Science and Medicine, Department of Neurology, University of Fribourg, Fribourg 1700, Switzerland

**Keywords:** interoception, attention, EEG, FND, movement disorder

## Abstract

The pathophysiology of functional neurological disorders (FND) has been discussed to include dysfunctions in interoception, the modality about perceiving and processing internal bodily signals. However, findings on abnormal interoception in FND have been inconsistent and mainly limited to measures of accuracy and self-report. Interoceptive neuronal markers have only been investigated in specific symptoms, and interoceptive attentional modulation has been completely overlooked. In a cohort of patients with mixed FND (*N* = 44) and sex- and age-matched healthy controls (*N* = 48), we set out to assess first, interoceptive accuracy with an adapted version of the heartbeat counting task; secondly, interoceptive self-report with two different questionnaires (Multidimensional Assessment of Interoceptive Awareness and Interoceptive Accuracy Scale) and thirdly, neuronal trait markers under attention modulation by measuring heartbeat-evoked potentials, the neurophysiological signal related to the heartbeat. We searched for group differences (FND versus controls) across two attentional conditions, by asking participants to either focus on their heartbeat (i.e. interoceptive condition) or an external sound (i.e. exteroceptive control condition). Cardiac covariates (heart rate and heartrate variability or normalized electrocardiac amplitude) were included in the analysis as control. Patients with FND scored lower in both interoceptive self-report questionnaires (*P* < 0.020), reported higher difficulty concerning the focus towards their heartbeat (*P* = 0.004), while no significant difference was found in interoceptive accuracy using the heartbeat counting task (*P* = 0.060). Global field analyses revealed short intervals of a group-by-condition interaction in global field power (285–298 ms) and topographical differences (310–321 ms) confirming that patients with FND have lower overall activity and frontal deactivation during the interoceptive condition. Preselected electrodes for a targeted analysis of the heartbeat-evoked potential based on earlier work revealed a medium effect size attenuation at the frontal-lateralized F8 electrode at 250–595 ms following R-peak for patients with FND (*P* = 0.028), surviving correction for cardiac covariates. Exploratory analyses further identified an earlier difference at F1 (185–210 ms post-R-peak) in FND patients for interoceptive attention (*P* = 0.001), also surviving covariate control. While behavioural interoceptive accuracy was marginally preserved, these findings indicate overall altered interoceptive processing in FND, characterized by reduced self-report and difficulty to focus on cardiac signals, along with attenuated neural processes, especially in frontal-lateralized regions that further depend on attentional mechanisms. By identifying objective neural markers of interoceptive dysfunction in FND, this study highlights the involvement of interoception in a multidimensional assessment including the relevance of attention.

## Teaser

Stoffel *et al*. report that patients with functional neurological disorders show altered brain responses when focusing on their heartbeat and experience greater difficulty in sensing bodily signals. These findings highlight altered neurophysiological patterns of interoception and suggest neuronal trait markers that might underlie the pathophysiology of this complex disorder.

## Graphical Abstract

**Graphical Abstract:**
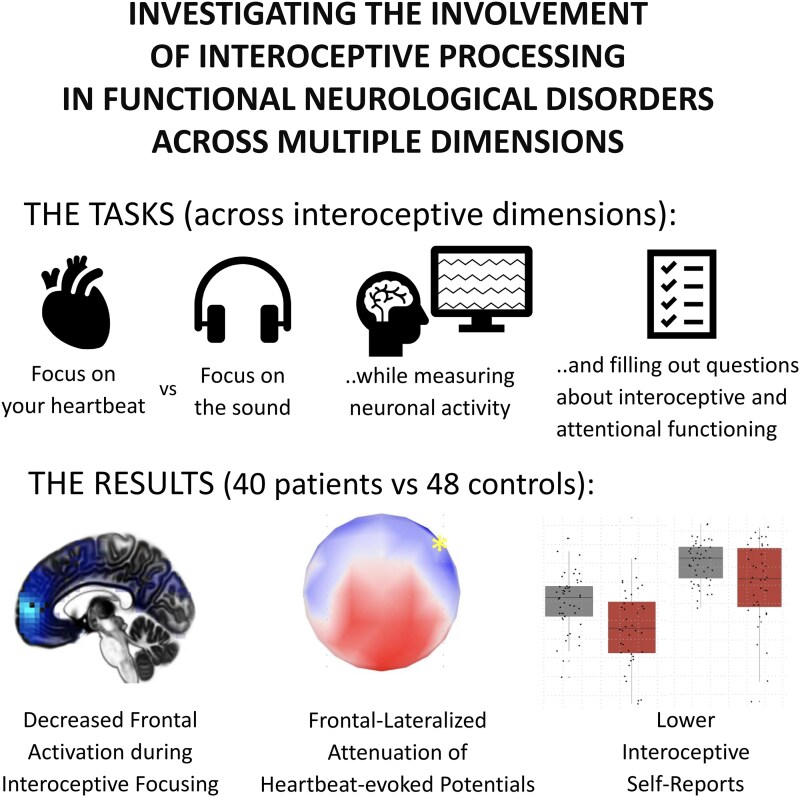

## Introduction

Functional neurological disorders (FNDs) are characterized by neurological symptoms including motor and sensory dysfunction or dissociative/functional seizures that arise from alterations in brain network functioning.^1^ Historically, it was hypothesized that these physical FND symptoms could represent a consequence of suppressed psychological stressors, following the Freudian model of conversion.^2^ More recent studies have clarified that the experience of trauma is neither necessary nor sufficient for the development of FND.^3,4^ Nonetheless, stressful and traumatic experiences are risk factors for FND, suggesting some involvement in the disorder’s pathophysiology.^5,6^ Recent advancements have further underscored the brain’s role in the active generation of emotional experiences through predictive modelling and inferences.^7^ Within this framework, emotions are not merely triggered responses to external stimuli, but context-dependent constructions shaped by prior experiences, internal bodily signals and conceptual knowledge. Applied to FND, this perspective suggests that disrupted construction of emotion categories combined with impaired interoception might contribute to FND symptom generation.^8,9^

Interoception refers to the sensing, integration and interpretation of bodily signals, at conscious and unconscious levels.^10^ The involvement of interoception, or interoceptive dysfunction, has been recently highlighted in FND research, in particular the theory of symptom formation through predictive coding frameworks and prediction errors based on low precision and salience attributed to interoceptive signals.^8,9,11,12^ Yet, research on interoception in FND has yielded heterogenous findings. Most work has measured cardiac interoceptive accuracy, defined as the behavioural accuracy or correct perception of the heartbeat.^13^ Some studies reported that patients with FND, compared with HCs, demonstrated lower interoceptive accuracy, assessed using the heartbeat counting or detection task (HCT).^14-17^ Other studies did not find differences between controls and patients.^18-20^ Notably, the validity of this traditional HCT has been criticized,^21,22^ and the heterogenous findings might be partly due to cognitive processes, informed guessing^23^ or differences in heart rate (HR).^24^ An attempt to improve the HCT’s validity included the addition of a breath-hold arousal, leading to heightened physical arousal which was associated with an increase of accuracy in a healthy population compared with psychiatric patients.^25^ Also, the adaptation to the instruction, motivating participants not to guess, could alleviate some of the criticism about the HCT in its traditional form, yet has only been implemented in two FND studies.^18,20^

Another investigated interoceptive dimensions is self-report, assessed using interoception questionnaires,^13^ pointing towards lower scores for FND patients.^15,17-19^ However, as different questionnaires have been used to assess this, the interpretation of the finding is still unclear, given each questionnaire, or their subscales, target different aspects of interoceptive self-report.^26^ Hence, it is crucial to test different questionnaires in the same population to target different aspects of this dimension, including the central aspect, using the multidimensional assessment of interoceptive awareness (MAIA)^27,28^ and self-reported accuracy (e.g. interoceptive accuracy scale; IAS^26,29^).

Next to these important and previously studied aspects of interoceptive accuracy and self-report, brain activity related to the processing of interoceptive signals has been proposed as an objective marker.^13^ In a recent study, aberrant neuronal processing in the cingulo-insular network was found for patients with FND, when focusing on and trying to count their heartbeats in an MRI setting.^30^ In another MRI study, 13 patients focusing on their interoceptive system (heartbeat, stomach or clinically affected body parts) showed an activation in the posterior cingulate cortex, the precuneaus as well as the caudate nucleus and the right insula compared with 13 HC and a control condition.^31^ While fMRI studies provide valuable spatial information, they may conflate different cognitive and perceptual processes due to their limited temporal resolution. This can be overcome by EEG-based measures such as the heartbeat-evoked potentials (HEPs).

HEPs reflect cortical responses to individual heartbeats in the EEG and represent a promising neural index of interoceptive processing. Recent meta-analyses indicate that HEPs are robust markers of interoception, modulated by attention, arousal and clinical status, showing moderate correlations with behavioural measures of interoceptive accuracy,^32^ which may serve as a neurophysiological trait signature of FND. A reduction in HEP amplitude over fronto-central regions has been observed in FND patients immediately prior to^33^ and during^34^ the onset of functional/dissociative seizures. However, no study to date has investigated HEPs in a large, mixed-symptom cohort of FND patients outside the context of acute seizure-like episodes. Such novel investigations could ideally uncover a broader interoceptive dysfunction in all subtypes of FND, independent of seizure onset.

Related to this, attentional processes are considered central to both FND pathophysiology and the modulation of HEPs. A recent review emphasized the importance of attentional quality and interpretative appraisal in interoceptive functioning.^35^ In healthy individuals, directing attention to the heartbeat was associated with increased HEP amplitudes at central electrodes,^36^ suggesting that HEPs are sensitive to top-down attentional focus. Yet, the interaction between attentional modulation and HEPs has not been explored in FND populations.

To address these questions, the present study investigates HEPs as a potential neuronal trait marker of interoception in a large, heterogeneous FND cohort, independent of acute symptom expression versus a control group, and assessed HEP responses during interoceptive (heartbeat-focused) versus exteroceptive (external stimulus-focused) attention. We integrate these HEP data with measures of an extended interoceptive accuracy task (HCT) and interoceptive self-report (MAIA and IAS). Additionally, by comparing brain responses during interoceptive versus exteroceptive attention, we aim to assess the modulatory role of attention on HEPs, thereby probing a central mechanism previously suggested to be implicated in FND. For that, we first applied an explorative approach into investigating potential sites, time intervals and overall global involvement of neuronal activation in this study, as it is the first state-independent analysis of attention-guided HEP assessment in FND. We complement the exploratory analysis by a targeted analysis based on preselected regions according to a previous study investigating functional seizure.^34^

## Materials and methods

### Participants

The study was conducted at the HFR as part of a larger project on interoception and biological markers (ClinicalTrials.gov NCT06084325; see previous publications^37,38^). Ninety-two participants were recruited for this study; four were excluded (three due to poor EEG quality, i.e. <70% of epochs accepted; one due to inability to complete the task), resulting in a final sample of *N* = 88 (95.65%) with for *N*_FND_ = 40 (75% female, mean age 39 years, SD = 11) and *N*_HC_ = 48 (73% female, mean age 38 years, SD = 13). Information on scholarity can be found in Supplementary Table 3. Patients with FND were recruited from two Swiss neurology clinics (University Hospital/Inselspital Bern and Cantonal Hospital of Fribourg; HFR), and age- and sex-matched HCs were recruited via flyers, online advertisements and word-of-mouth. The inclusion criteria were FND diagnosis (F44.4–44.7 ICD-11) for patients, age ≥18 years. The exclusion criteria were major psychiatric or neurological disorders, previous brain surgery, medical implants, substance abuse, cardiovascular disease and pregnancy or breastfeeding.

The study was approved by the Ethics Committee of Canton Bern (2023-00469) and conducted in accordance with the Declaration of Helsinki. Written informed consent was obtained from all participants.

### Demographic and clinical characteristics

Before the study visit, participants completed questionnaires on somatoform dissociation (SDQ-20),^39^ depression (BDI-II)^40^ and anxiety (STAI).^41^ Due to high correlation between BDI-II and STAI-T (*r* = 0.77, *P* < 0.001), a composite score of affective symptoms score was computed for further analysis by adding the BDI-II and STAI-T score. Medical diagnoses, medication use and for females, menstrual cycle or hormonal contraceptive status were recorded. FND patients additionally underwent standard neurological assessment, including the SFMDRS^42^ and the Clinical Global Impression Scale (CGI)^43^ for symptom severity. For details on study design, see Stoffel *et al*.^37^

### Interoceptive self-report and accuracy

Interoceptive self-report was assessed prior to the study via the MAIA-II^28^ for a MAIA_TOTAL_ sum score, along with the IAS.^29^ Interoceptive accuracy was assessed using an adapted HCT. In the neutral condition, participants counted perceived heartbeats during three randomized intervals (25 , 35  and 45 s) without checking their pulse.^44^ Instructions emphasized perception over estimation, allowing zero responses if no heartbeat was felt (as in Millman *et al*.^18^ and Sojka *et al*.^20^). After each trial, participants rated confidence, intensity and difficulty (0–100 scale). Additionally, participants repeated the HCT while being instructed to hold their breath for as long as they could tolerate to increase physiological arousal (as in Smith *et al*.^25^). HR was measured using electrocardiography (ECG). Each participant completed six trials (three per condition), and accuracy per trial was calculated as 1 − ([(recordedheartbeats−countedheartbeats)/  recordedheartbeats]), then averaged across trials.^44^ The mean accuracy was computed per condition and across both conditions.

### Heartbeat-evoked potential and attentional modulation

After completion of the HCT, neural responses to interoceptive signals were assessed via HEPs, derived from EEG signals time-locked to ECG R-peaks. For the measurement of HEP, we followed the set-up of Petzschner *et al.*,^36^ instructing participants to direct their attention to either their heartbeat (interoceptive condition) or the continuous white noise (exteroceptive condition), each repeated 10 times for 20 s. Auditory stimuli (i.e. white noise) were delivered through in-ear headphones in both conditions. After completion, participants rated perceived ability, difficulty and intensity for each condition. Full task details are provided in the Supplementary Fig. 1.

### EEG and ECG acquisition and preprocessing

EEG was recorded using a 64-channel BIOSEMI ActiveTwo system (Biosemi, Amsterdam) with an occipitally placed CMS-DRL ground at sampling rate of 2048 Hz. ECG was recorded via four electrodes placed diagonally across the torso, taking signal from the right clavicle to the left rib cage for R-peak detection, while the second pair served as a backup.

R-peaks were visually verified after offline detection using the Chernenko MATLAB script.^45^ For the ECG, heartbeat-locked responses were computed after mean-centring, band-pass filtered between 0.3 and 40 Hz and averaged epochs from −100–652 ms around the R-peak. EEG data preprocessing was performed offline using a customized MATLAB toolbox (EEGpal), based on EEGLab 2023.1,^46^ available at https://github.com/DePrettoM/EEGpal. Data were band-pass filtered (0.3–40 Hz),^47^ and independent component analysis^48^ was used to remove artefacts attributed to eye movements,^49^ removing on average 1.58 components (SD = 0.84) per participant. Bad channels were interpolated using the spherical splines,^50^ averaging 1.18 electrodes (1.85%) per participant. Data were re-referenced to the average, and ERPs were computed averaging epochs from −100–652 ms around the R-peak, without baseline correction.^36^ Epochs exceeding 100 µV in any channel were rejected, excluding of three participants (>33% rejected epochs) and yielding a final mean acceptance rate of 95.42%.

### Statistical analysis

For behavioural data, *t*-tests were used for parametric data, the Wilcoxon rank-sum test for non-parametric data, and the χ^2^ or Fisher’s test for binary variables when calculating a group difference. *P*-values and Cohen’s *d*, or odds ratio (OR), respectively, are reported for these analyses. False discovery rate (FDR) correction was applied for multiple comparisons. Correlation analyses were done using Pearson correlation, and association analyses were calculated using a linear model including covariates of no interest. HR was operationalized via the mean distance in milliseconds between two detected R-peaks. Heartrate variability (HRV) was calculated using the root mean square of successive R–R interval differences (RMSSD).^51^

Where we ran an ANOVA, Shapiro–Wilk’s test for normality and Levene’s test for homogeneity of variance were conducted to test the assumptions of the ANOVA. Given ANOVA’s robustness to mild deviations of non-normality, and with an adequate sample size per group in a balanced design, we proceeded with ANOVA analysis, even if normality assumptions were not met.^52^ Nonetheless, we then also applied the aligned rank transform (ART) as an additional method to investigate whether the main group effects remain when conducting a non-parametric factorial ANOVA.^53^

Wherever applicable, significant effects are reported with *F*-values, *P*-values and effect sizes using Cohen’s *f*.

### EEG analysis

For EEG, we report significant results of a minimal duration of 10 ms and considering time periods starting as early as 150 ms following the R-peak, which has been reported to be an early neuronal response to the heartbeat.^54,55^ The exploratory inspection of neurophysiological differences between groups and condition lead to the decision of also reporting such small time intervals; however, these findings should also be interpreted with caution.

#### Global field power and topographical analysis

First, to test overall brain activity and topography at a global level (i.e. across the entire scalp) in an unbiased and data-driven method, we performed a statistical analysis for the two-factorial design of group and condition using the RAGU toolbox. RAGU performs a assumption-free randomization analysis (bootstrapping) for the multichannel neurophysiological data.^56^ This toolbox has been used before to test overall neurophysiological differences in clinical groups across two conditions.^57-59^ This statistical approach allows to identify changes in signal strength modulation, the global field power (GFP), as a main effect of group or in interaction of group and condition. Also, RAGU allows an overall analysis of changes in topographical modulation, using a topographical ANOVA (tANOVA) to identify different brain networks that are activated in each specific moment. For this, L2 normalization was applied for the significant results to reflect only the topographical difference and signal strength. For analyses in RAGU, the *P*-threshold was set at 0.05 and the analyses were computed with 5000 runs, reporting significant effects with the overall explained variance by this factor(s).

#### Source localization

Group and interaction effects identified at the global level (GFP or tANOVA) were further explored at the source level using voxel-wise independent or paired *t*-tests on sLORETA-estimated (standardized low resolution brain electromagnetic tomography) current density distributions.^60^ The source analysis was implemented in time windows previously found to be statistically significant, serving primarily to visualize the cortical origins of these identified effects rather than conducting an independent statistical test. Source-level comparisons were carried out on untransformed data with a signal-to-noise ratio set to 100. Subject-wise normalization was applied, without baseline correction, and statistical testing was limited to a single average comparison within each time window. To control the family-wise error rate associated with multiple voxel-wise comparisons when running the tests, non-parametric permutation approach with 5000 iterations was used to derive corrected thresholds for significance.

#### Heartbeat-evoked potential analysis

##### Explorative analysis on all traces

First, to complement the explorative global analysis also at trace-level, a group comparison was ran including all electrodes and the full epoch length. Here, an unpaired randomization test with 5000 iterations was calculated to identify any electrodes as a local difference between groups separately per condition. FDR was used to correct for multiple comparisons. These results are presented in detail in the Supplementary material.

##### Targeted traces on Fz, F7 and F8 between group

Secondly, a targeted trace analysis was conducted, selecting electrode locations based on previous research of a within-group analysis (F8, Fz and F7 at 250–595 ms after R-peak).^34^ Individual amplitudes for these three electrodes for the defined time interval were extracted per participant and condition via EEGpal. Repeated-measures ANOVA were conducted with condition as a within-subject factor and group as a between-subject factor, using subject as an error term to account for within-subject dependencies. We also ran control analysis, with electrode as an additional site factor, similar to Flasbeck *et al.*^34^ This was implemented to identify regional specificity for the HEP attenuation in FND compared to HCs, and how this might be influenced by the attention-guiding condition. Detailed methods and results can be found in Supplementary material.

##### Targeted traces on Fz, F7 and F8 separate for condition

Finally, group comparisons were done separately per condition, investigating exploratively the attentional modulation as a difference between groups. For this, trace analyses were done in Cartool^61^ reporting intervals for these three electrodes with *P* < 0.05 after correcting for multiple comparison.^61^

### Correlation and control analysis

We extracted the GFP amplitudes and the mean values of the amplitudes of the identified electrodes at the local trace-level per participant for the identified intervals using EEGpal. To perform correlation analysis on topographical results, global map dissimilarity (GMD) was computed to obtain a single measure of topographical change over time per condition, also using EEGpal. These values were then used to run further correlation analyses with other interoceptive or clinical variables collected within the study or used to run linear or linear mixed-effect models, where we can control for covariates like sex, age, along with cardiac (HR and HRV) and psychiatric variables (intake of medication and affective sum score). To guarantee no cardiac interference with the identified EEG effects, we extracted the amplitude of the normalized ECG trace for the respective time intervals and included this as a control variable when running linear models.^34^ Normalized ECG traces were obtained by dividing the heartbeat-locked response by the range of its signal (maximum–minimum amplitude) for each participant. Also, ratings on difficulty of focusing on the interoceptive or the exteroceptive control condition were used as a control variable.

## Results

### Demographic and clinical characteristics

The two groups did not differ in terms of age or sex [*t*(40.74) = −0.01, *P* = 0.99 and χ^2^(1, *N* = 88) = 0.00, *P* = 1.00, respectively]. As expected,^62^ patients with FND reported higher scores in depression (*d* = 1.36, *P* < 0.001) and anxiety (trait: *d* = 0.80, *P* < 0.001 and state: *d* = 1.04, *P* < 0.001) compared to controls. Unsurprisingly,^63^ patients with FND also reported a higher intake of psychotropic, antiepileptic, opioid and benzodiazepine medications (χ^2^ = 21.09, *OR* = 16.26, *P* < 0.001). Patients with FND had a higher HR compared to controls (*d* = −0.72, *P* = 0.001) and a lower HRV (*d* = −0.78, *P* < 0.001) representing enhanced sympathetic activation, as previously reported in FND (Table 1).^34,64,65^ For further information on clinical characteristics and comorbidities see Supplementary Tables 1 and 2.

**Table 1:** Demographics

Variable	Overall, *N* = 88	HC, *N* = 48	FND, *N* = 44	*P*-value
Female sex, count (%)	65.0 (73.9)	35.0 (72.9)	30.0 (75.0)	>0.9^a^
Age in years, mean (SD)	38.4 (12.2)	37.8 (13.0)	39.1 (11.3)	0.6^b^
Heartrate R–R interval in ms, mean (SD)	882.5 (127.0)	921.2 (126.1)	834.9 (112.3)	0.001^b^
Heartrate variability RMSSD in ms, median (IQR)	36.0 (22.2)	41.0 (27.0)	28.1 (16.6)	<0.001^c^
Psychotropic/antiepileptic/opioid/benzodiazepine medication, count (%)	22.0 (25.6)	2.0 (4.3)	20.0 (50.0)	<0.001^d^
Depression, median (IQR)	8 (12)	4 (7)	15 (13)	<0.001^b^
State anxiety, median (IQR)	32.0 (14.3)	28.0 (8.5)	38.5 (20.0)	<0.001^b^
Trait anxiety, mean (SD)	41.3 (11.9)	37.3 (10.3)	46.1 (12.0)	<0.001^c^

^a^χ^2^ test; *N* (%).

^b^Two-sample *t*-test; mean (SD).

^c^Wilcoxon rank-sum test; median (IQR).

^d^Fisher's exact test; *N* (%).

### Lower interoceptive self-report and no difference in accuracy

**Figure 1:**
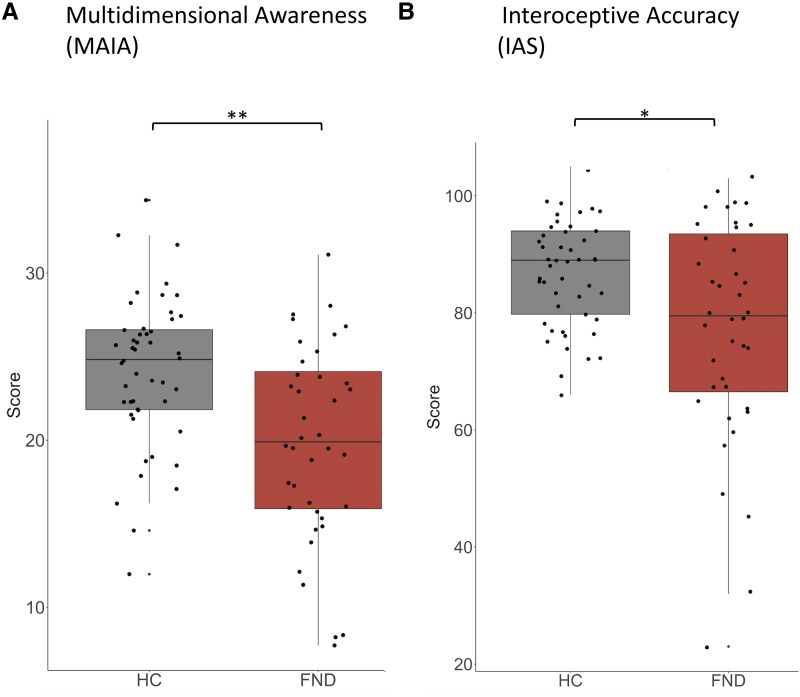
**Differences across groups in interoceptive self-reports.** Differences across groups (HC = 48 healthy controls compared with FND = 44 functional neurological disorders) for (**A)** MAIA_TOTAL_ = multidimensional assessment of interoceptive awareness using a parametric *t*-test with *P* = 0.002 and (**B)** IAS = interoceptive accuracy scale using a non-parametric Wilcoxon test with *P* = 0.020. Each data point represents a participant.

As previously reported in a publication discussing respiratory interoception in the same cohort,^38^ patients reported lower scores than controls on both interoception questionnaires (MAIA_TOTAL_: *d* = −0.73, *P* = 0.002 and IAS: *d* = −0.67, *P* = 0.020), Fig. 1. For the MAIA, the specific subscales of ‘Trusting’ (*P* = 0.005), ‘Self-Regulation’ (*P* = 0.005), ‘Emotional Awareness’ (*P* = 0.043) and ‘Attention Regulation’ (*P* = 0.043) showed a lower score for patients after adjusting for multiple comparison with FDR (Supplementary Fig. 2).

In the dimension of interoceptive accuracy, there was marginal evidence for a difference in the adapted HCT between groups (mean: *d* = −0.43, *P* = 0.060). Assessing the confidence of their performance as another measure of self-report,^13^ there was no group difference. Finally, there was no change in accuracy or confidence between the neutral and breath-holding arousal condition for either group. For an overview of HCT results see Supplementary Table 4.

### Global field power: weaker frontal activation in the interoceptive condition in FND

**Figure 2:**
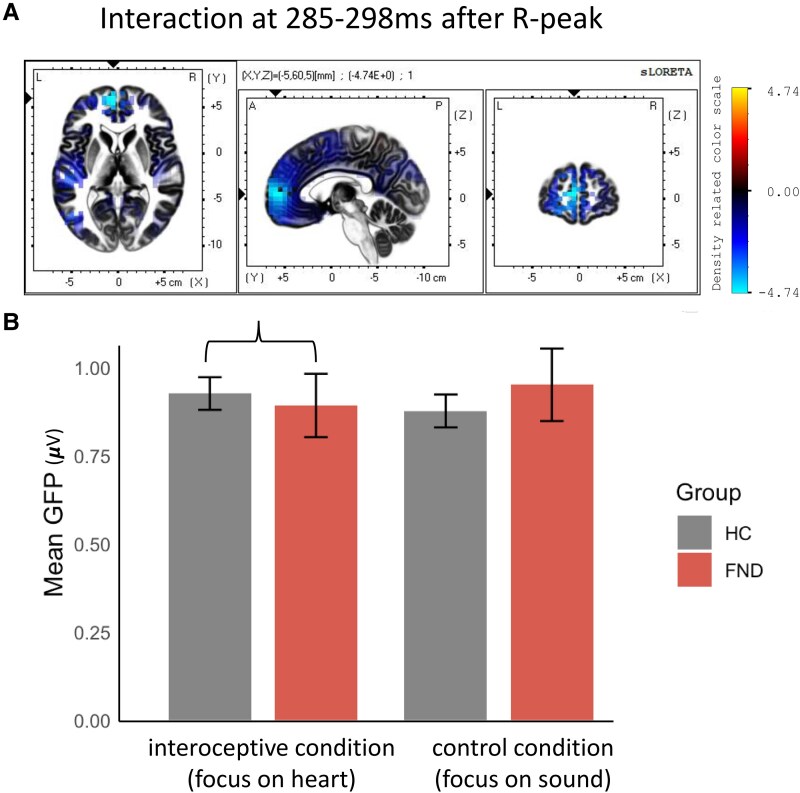
**Interaction of group and condition at 285–298 ms after R-peak and source localization for the difference in the interoceptive condition.** (**A)** The interoceptive-specific differences across groups indicates less activity in the interoceptive HEART condition for *N* = 40 patients with FND compared with the *N* = 48 HC at Brodmann area 10 (aPFC), medial frontal gyrus and frontal lobe (sig after testing for multiple comparison using a two-tailed independent *t*-test in sLORETA, (*t* = −4.74, *P* = 0.002). (**B)** The identified significant time interval for an interaction effect at 285–298 ms after R-peak (derived from the randomization analysis in RAGU). GFP bars illustrate mean and standard error for both groups and conditions.

Using a 2 × 2 factor analysis in RAGU, we were not able to identify a main effect of group (*P* = 0.647), but a hint of a significant interaction of both factors at 285–298 ms after R-peak (13 ms; *P* = 0.027 with mean explained variance of 5.0%). Using linear mixed-effect models with restricted maximum likelihood on the extracted data, this interaction remained when controlling for covariates such as sex, age or cardiac differences or affective symptoms and medication [*b* = 0.11, SE = 0.049, *t*(86) = 2.20, *P* = 0.030], demonstrating lower GFP in the interoceptive condition for FND. Using sLORETA to localize the source of this effect, a lower activation in anterior prefrontal cortex (aPFC, Brodmann area 10) and the medial frontal gyrus for the interoceptive condition in patients with FND compared to HC was identified, Fig. 2.

### tANOVA: altered topographies reflecting distinct frontal activation

**Figure 3:**
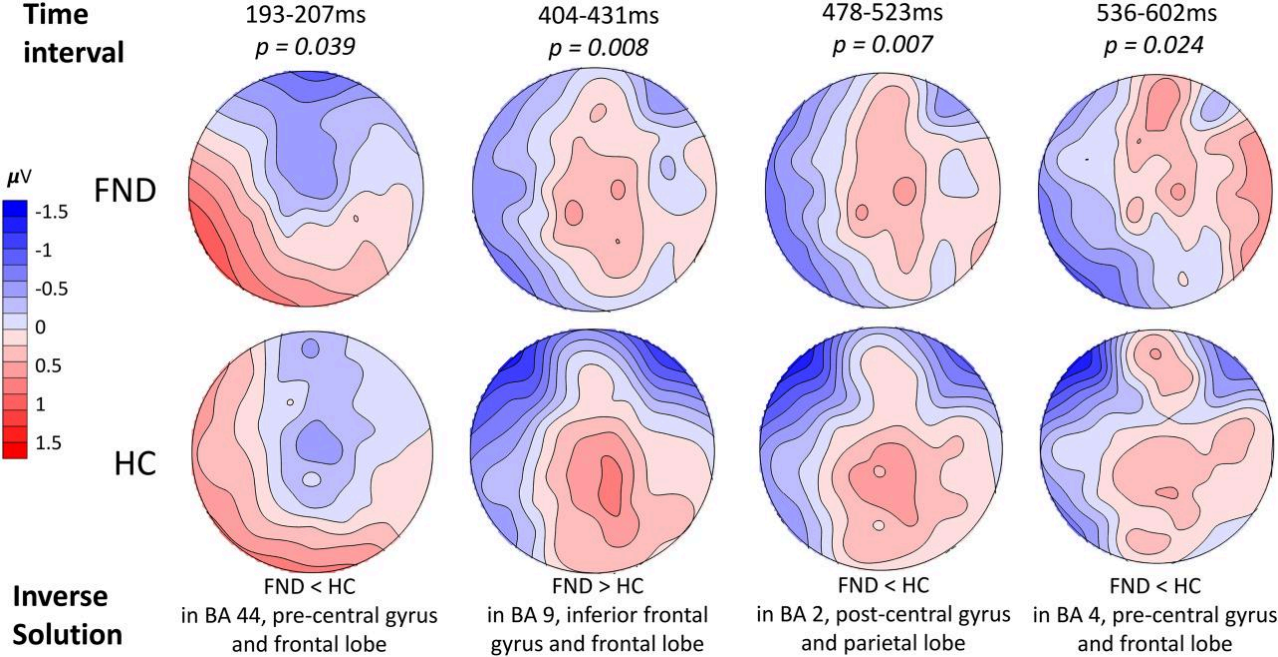
**Different topography between groups in the attention task (including both conditions of interoceptive and control condition).** Time intervals indicate significant differences in topography during the time intervals of milliseconds after R-peak, at the level of *P* < 0.05. Source localization was computed using randomization tests in sLORETA, identifying various Brodmann areas (BA) as estimated origin of the differential activation between the two groups of FND (*N* = 40) and HC (*N* = 48).

Topographical difference analysis revealed a main effect of group in four different time intervals [193–207 ms (14 ms) with *P* = 0.039 and explained variance = 2%; 404–431 ms (27 ms) with *P* = 0.008 and explained variance = 2.3%; 478–522 ms (44 ms) with *P* = 0.007 and explained variance = 2.4% and 536–602 ms (66 ms) with *P* = 0.024 after R-peak; explained variance = 2.3%]. The respective topographies and the inverse solution identifying the differential activation in neuronal networks are shown in Fig. 3. A main effect of condition at 510–524 ms following R-peak was detected, suggesting the study design did indeed lead to the activation of different neuronal networks depending on the attentional focus. The findings of condition are described in Supplementary Materials.

**Figure 4:**
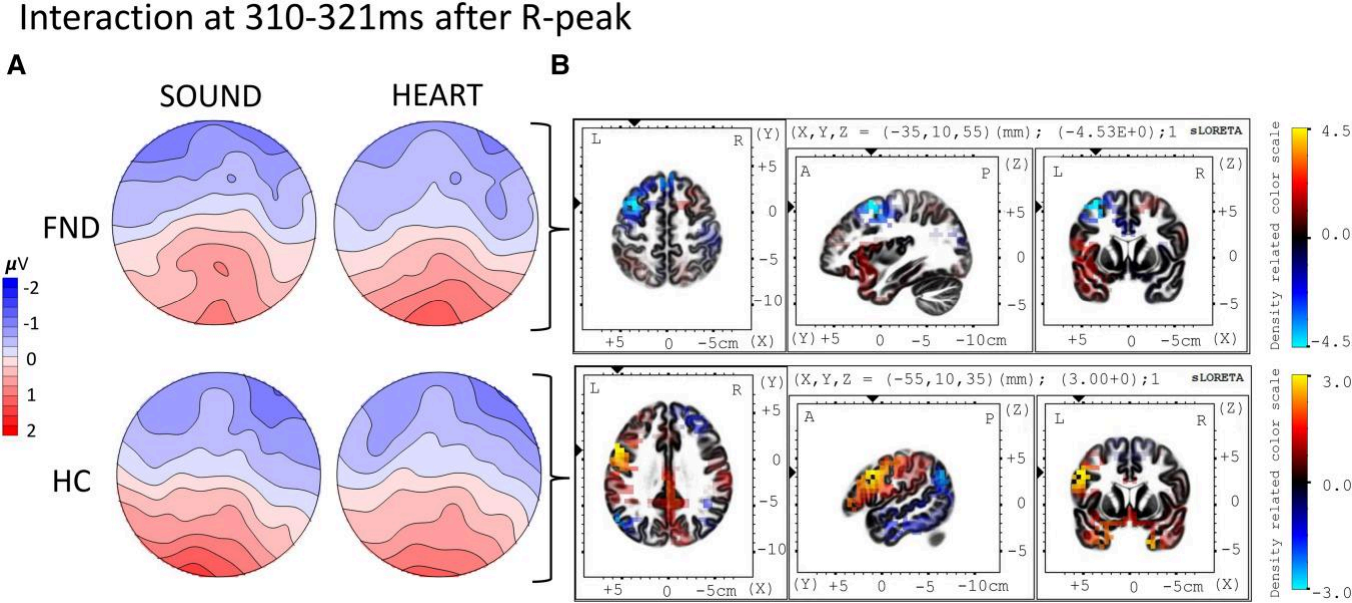
**Interaction of group and condition at 310–321 ms post-R-peak and respective source localization.** (**A**) Topographies represent the identified significant time interval for an interaction effect at 310–321 ms (significant after randomization testing in RAGU). (**B)** Group-specific within-group comparison of condition using inverse solution with sLORETA indicating less activity in the interoceptive HEART condition compared with the control SOUND condition for patients with FND (*N* = 40) in Brodmann area 6, the middle frontal gyrus and the frontal lobe (significant after testing for multiple comparison from performing 5000 randomizations in a within-subject *t*-test; *t* = −4.53, *P* = 0.009. The condition-specific difference of topography in the healthy control group (HC, *N* = 48) identified increased activity for the interoceptive HEART compared with the SOUND condition at Brodmann area 9, the inferior frontal gyrus and the frontal lobe (*t* = 3.00, *P* > 0.05 after controlling for multiple comparison).

We identified a hint for an interaction between group and condition at 310–321 ms post-R-peak (11 ms) with *P* = 0.019; explained variance = 2.4%. Using sLORETA, we identified that patients with FND demonstrate a deactivation of the middle frontal gyrus and adjacent parts of the frontal lobe (BA6) during this time interval (two-tailed *t*-test with *P* = 0.009 after adjusting for multiple comparisons) for the interoceptive condition, compared to the control condition, Fig. 4. Activation in HCs differed and showed activation of more ventral parts of PFC, centring on inferior frontal gyrus and adjacent parts of frontal lobe (BA9) for the interoceptive compared to the control condition, marking the topography at 310–321 ms (*P* > 0.05).

### HEP: altered frontal traces in FND

#### Targeted analysis between group

EEG analysis revealed a prominent HEP at frontal electrodes, similar to previous HEP work.^66-68^ Testing each of the three electrodes (Fz, F7 and F8) in the time interval of 250–595 ms following R-peak,^34^ we identified a group effect for F8: *F*(1, 86) = 5.46, *P* = 0.022, *f* = 0.25, indicating a medium-sized group difference. It should be noted that the Levene’s test confirmed homogeneity of variances across groups (*P* = 0.945), yet Shapiro–Wilk tests indicated violations of normality in two out of four conditions (interoceptive condition in HC and control condition FND). When implementing the ARTool analysis, which is robust for violations of normality, the group effect was further confirmed [*F*(1, 86) = 5.62, *P* = 0.020]. This group difference remained significant when adding covariates of no interest [sex and age; *F*(1,84) = 5.76, *P* = 0.022, or cardiac differences with HR and HRV; *F*(1, 84) = 5.40, *P* = 0.023], or ECG amplitude; *F*(1, 85) = 5.72, *P* = 0.019), or psychiatric differences with intake of medication and affective symptoms; *F*(1, 84) = 5.42, *P* = 0.022).

However, the group effect was also present when running a non-parametric ART analysis including site as a factor for all three electrodes of Fz along with the strongly lateralized F7 and F8 electrodes [*F*(1, 86) = 5.50, *P* = 0.021]. A control analysis using alternative sites (Fz with less lateralized F3 and F4) revealed also a group effect [*F*(1, 86) = 6.14, *P* = 0.015]. An interaction with condition in this control analysis was found for the central control region (C3, Cz and C4), suggesting an FND-specific difference of EEG traces dependent on the attention mechanisms. More details in Supplementary material.

#### Targeted analysis separate for condition

**Figure 5:**
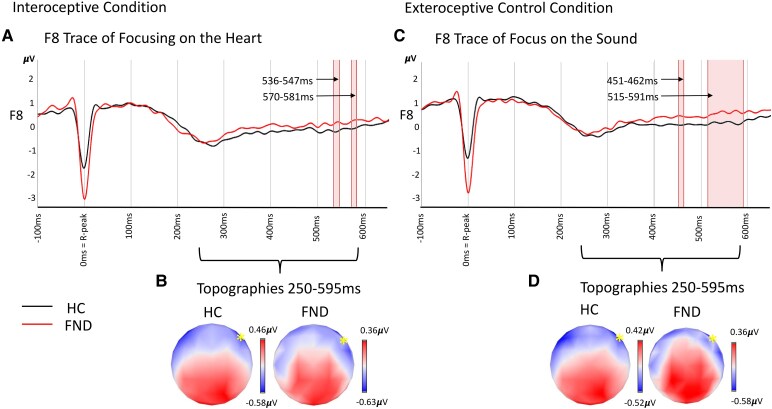
**HEP illustration per group and condition.** Grand average for electrode F8 associated with the reduced HEP for *N* = 40 patients with FND (red) and *N* = 48 HC (black) with the time-axis representing ms from R-peak. (**A)** The trace for the interoceptive condition of focusing on the heart with (**B)** for the corresponding topographies for 250–595 ms after R-peak and trace F8 marked as a yellow asterisk (no statistical test, simply for descriptive illustration of topography during the full time interval per group and condition). (**C)** The trace for the control condition of focusing on the sound with (**D)** for corresponding topographies, respectively (again descriptively as illustration). However, the red highlighted area in (**A**) and (**C**) represents the time interval within the investigated 250–595 ms interval, where F8 became significant (*P* < 0.05) in the group comparison separately per condition using a between-group randomization test.

Analysing the averaged HEP at electrodes F8 separate for conditions, we found group differences in two time windows for each condition (interoceptive: 536–547 and 570–581 ms after R-peak and control: 451–462 and 515–591 ms after R-peak), Fig. 5. All group differences survived the control for sex and age. Testing then further covariates in the model, and controlling for multiple testing, all time intervals but F8 451–462 ms in the control condition survived for the control of cardiac differences (HR and HRV), while no group difference except F8 at 515–591 ms in the control condition survived the control for affective symptoms and medication (*P* = 0.040).

Finally, for F7 and Fz, we did not identify a group difference, Supplementary Fig. 3.

#### Explorative analysis

Performing a randomization test between groups in the interoceptive condition for all traces revealed an enhanced and earlier negativity in patients with FND at F1 in 185–210 ms after R-peak, surviving correction for cardiac and psychiatric covariates (Supplementary Fig. 4). For the control condition, two later time intervals were identified at electrode F8 at 540–550 ms and at electrode P6 for 620–630 ms after R-peak. These results are further discussed in Supplementary material.

### Correlation analysis: interoceptive and clinical variables

None of the identified neurophysiological group difference would be associated with interoceptive variables and survive correction for multiple comparisons. The effect of condition [GMD of 510–524 ms (14 ms) following R-peak] was positively associated with self-rated somatoform dissociation scores in FND (*r* = 0.55, *P*_adj_ < 0.001), as well as with HCT (*r* = −0.25, *P*_adj_ = 0.034), Supplementary Fig. 5. We did not identify any further correlations between clinical variables and the extracted HEP scores.

### Control analysis: attentional difficulty and cardiac differences

Patients with FND reported a higher difficulty focusing on their heart compared with controls (*d* = −0.49, *P* = 0.004 after correcting for multiple comparisons). For the perceived intensity of the heartbeat or sound, and the ability to focus on them, there were no differences between groups (Supplementary Table 5).

**Figure 6:**
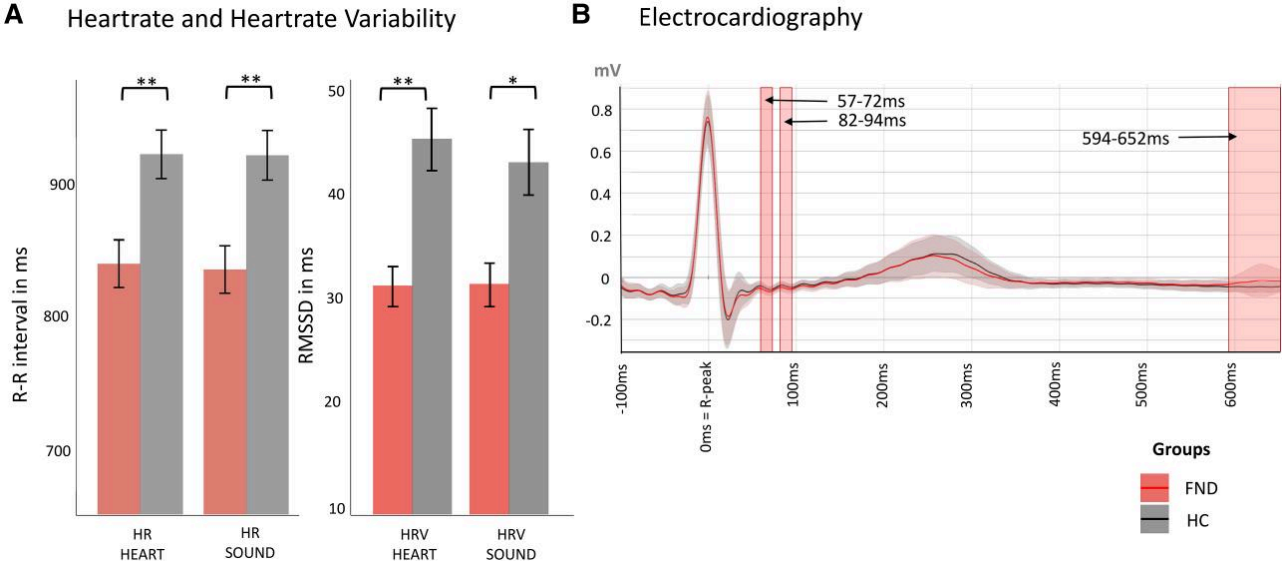
**HR and HRV separately per group and condition.** (**A)** Box plots showing within- and between-group tests (*N* = 40 patients with FND and *N* = 48 HC. HR was operationalized by the time in milliseconds between the two offline detected R-peaks in the ECG, i.e. R–R interval in milliseconds. Mean values and associated bpm are FND_SOUND_ = 836 ms (71.8 bpm), FND_HEART_ = 840 ms (71.4 bpm), HC_SOUND_ = 921 ms (65.2 bpm), HC_HEART_ = 923 ms (65.0 bpm). The HRV was operationalized by the RMSSD (in ms) illustrating mean values for FND_SOUND_ = 31 ms, FND_HEART_ = 31 ms, HC_SOUND_ = 43 ms, HC_HEART_ = 45 ms. Significance levels of the repeated-measures ANOVA performed: ns: *P* > 0.05, *P < 0.05, **P < 0.01, ***P < 0.001, after correcting for multiple comparisons using FDR. (**B)** ECG signals were baseline-corrected using the average over their entire duration (−100–652 ms around R-peak). ECG traces were normalized according to their amplitude range (maximum minus minimum) to correct for differences in amplitude due to electrode placement. Traces, along with standard deviations, are shown separate per group with portraying in red shaded time periods significant for a group difference at the level of *P* < 0.05 using a two-sample *t*-test and correcting for multiple comparisons.

For the cardiac control, the repeated-measures ANOVA detected a main effect of group for both the HR and the HRV [*F*(1, 86) = 10.51, *P* = 0.002, *f* = 0.35 and *F*(1, 86) = 12.33, *P* < 0.001, *f* = 0.38, respectively], while the condition (*P* = 0.058, and *P* = 0.323, respectively) and the interaction term in these models (*P* = 0.142, *P* = 0.298, respectively) did not meet the threshold of significance, Fig. 6A. Note that for the HR, Shapiro–Wilk tests indicated normality (*P* > 0.38), and Levene’s test confirmed homogeneity of variances (*P* = 0.542), satisfying assumptions for ANOVA. For HRV however, both homogeneity of variances (Levene’s test: *P* = 0.012) and normality in three out of four combinations (Shapiro–Wilk tests: *P* < 0.05) was violated. However, the ARTool analysis robust for violating these assumptions confirmed the group effect with *F*(1, 86) = 10.79, *P* = 0.002. Finally, inspection of the normalized ECG trace (accounting for potential individual difference in amplitudes due to electrode placement) revealed two early and a late group differences, but none of these fall into our interval of interest (i.e. 150–595 ms after R-peak), Fig. 6B. Nonetheless, ECG amplitude were extracted for time intervals where we identified global or trace differences (i.e. 185–321, 285–298, 310–321 ms and the full epoch of 250–595 ms), demonstrating no group differences (*P* > 0.124). The group differences and interactions also remained when adding the ECG amplitude of the respective time interval in the models as a covariate.

Investigating whether the differences in self-rated difficulty to focus during the interoceptive or the control condition, or the differences in cardiac physiology were correlated to the detected HEP differences, we ran correlation analyses with these control variables and our main outcome variables.^33^ Neither of the significant time intervals of identified neuronal markers for a group differences detected (i.e. F1 at 185 ms, F8 at four time intervals, GFP at 285 ms and GMD at 310 ms) was correlated with the self-rated difficulty in interoceptive condition (*P* > 0.311), the HR (*P* > 0.222) nor the HRV (*P* > 0.271), suggesting the difference at the level of brain processing was unrelated to self-reported difficulty to focus or underlying cardiac rhythm differences.

## Discussion

Aiming at investigating comprehensively interoception in a mixed cohort of patients with FND, we assessed interoception at the level of self-report, accuracy, confidence ratings and neural mechanisms based on attention-guiding. Concerning the two latter, we explored state-independent brain activity of interoceptive processing (HEPs), conducting explorative analysis, in addition to predefined frontal regions. In our study, we observed reduced interoceptive functioning across multiple dimensions: patients reported lower interoceptive self-report (measured via MAIA and IAS), preserved normal interoceptive accuracy (measured via the HCT) and reduced neural activations, specifically in the lateralized, frontal regions (measured via reduced HEP in mid-frontal regions and in particular electrode F8). FND patients also self-reported greater difficulty focusing attention on their heartbeat, and demonstrated altered frontal HEPs associated with interoceptive focus (F1). Together, these findings support a multidimensional disruption of interoception in FND, encompassing self-report, attentional aspects and neurophysiological correlates, while interoceptive accuracy remained normal.

### Lower interoceptive self-report and preserved heartbeat counting

Patients with FND reported reduced multidimensional awareness (MAIA_TOTAL_) and lower interoceptive accuracy (IAS) compared with controls. While the total score of the MAIA is central,^27^ its subscales also capture distinct aspects of it.^69^ In our cohort, ‘Trusting’, ‘Self-Regulation’, ‘Emotional Awareness’ and ‘Attention Regulation’ were particularly reduced, consistent with priors reports on lower ‘Trusting’ and ‘Not-Distracting’ in other FND populations.^18,19^ Notably, also the aspect of interoceptive accuracy was reduced (assessed via the IAS), revealing that both of the two distinct aspects of self-reported interoception are diminished in FND.^26^

In contrast, no group difference was observed in HCT accuracy, though a moderate effect was present (effect size of *d* = −0.43 with *P* = 0.060). While some studies have before reported reduced HCT scores in FND,^15-17,33^ others did not.^18-20,70^ Importantly, all previous studies had smaller sample sizes (reaching from overall *N* = 33 to *N* = 76), underscoring the improved generalizability of our findings. Moreover, we implemented pooled trials across two different conditions (six in total), unlike most prior studies using only three (except Sojka *et al*.^20^). Also, we followed novel best practices to reduce guessing (along Millman *et al.*^18^ and Sojka *et al*.^20^), to prevent a gain in accuracy via non-interoceptive components.^21,23^ As the absence of group differences persisted even with the addition of a breath-hold arousal condition, these findings extend and align with previous reports showing no difference in the HCT in patients with FND.^18-20,70^

### Attentional deficits in interoceptive processing

The short identified group-by-condition interaction in global brain activation ∼300 ms post-R-peak suggest altered interoceptive attention in FND. This window coincides with diastole, when interoceptive noise typically subsides to optimize signal integration.^71^ In FND, however, previous work suggests a failure to suppress such noise, indicated by the impaired implicit cardiac modulation effect in patients with functional/dissociative seizures.^72^

In our cohort, FND patients showed briefly, a reduced GFP and a deactivation of frontal regions during interoceptive focus between 285 and 298 ms (14 ms) compared with HCs, whose results pattern are more consistent with prior studies reporting HEP enhancement with internal attention.^54^ Notably, this failure to enhance overall HEP amplitude in interoceptive focus also appears in depersonalization/derealization disorder.^73^ Source localization revealed reduced engagement of two frontal regions (e.g. aPFC and medial frontal gyrus) during interoceptive attention in FND. These regions are involved in attentional control,^74^ salience processing^75^ and interoceptive monitoring^76^ and accordingly, this decreased activation may reflect a failure to tag interoceptive signals as salient, potentially contributing to subjective difficulty in accessing internal bodily states. No direct correlation emerged between neural activation and subjective difficulty ratings, and no group differences were observed in perceived heartbeat intensity. This suggests that altered neural recruitment may precede and shape subjective and the self-reported experience, rather than simply reflect it.

Additional short group-by-condition interactions at 310–321 ms (11 ms) also indicated altered brain network activation for interoceptive processing: FND patients showed decreased activation in premotor cortex, while controls exhibited increased activity in anterior and lateral regions of the prefrontal cortex. These patterns are consistent with reduced prefrontal engagement in FND during interoceptive versus exteroceptive focus^30^ and mirror stress-related frontal dysregulation observed in other psychiatric conditions.^77,78^ The absence of prefrontal recruitment in FND thus may indicate that interoceptive focus is experienced as stressful, in line with the role of stress, arousal and trauma in FND aetiology.^5,6^

### Attenuation of frontal HEP at F8

Our targeted analysis further revealed attenuated late HEP amplitudes at frontal-lateralized electrode (F8; 250–595 ms post-R-peak) in FND, extending earlier findings in functional/dissociative seizures patients to both a larger and a broader FND population, but also independent of symptom onset.^33,34^ This effect suggests a stable neurophysiological alteration in frontal-lateralized interoceptive processing in FND. Our finding on the right side is in line with evidence discussing a functional lateralization for cardiac interoceptive signal integration.^79,80^ However, in the control analysis for regional specificity, also the less lateralized choice of electrodes (F3, F4 along with Fz still) shows a main effect of group. Nonetheless, the attenuation of the frontal-lateralized F8 in FND is supported also by the explorative data-driven approach. Yet, this was only the case in the control condition. Note that there was no significant interaction effect, yet the specificity in condition, derived from identifying the F8 electrode as a group effect only in the control condition in the global trace analysis, could still point towards the fact that the attentional guiding towards the heartbeat could enhance interoceptive processing across group (as expected^36^). Concerning the absence of identified attenuation in the frontal-central sites (as in the previous study on functional/dissociative seizures^34^), it might be a state or symptom subtype-specific effect, or it could be due to differences in methodology. However, the control analysis conducted similarly to this study also identifies an FND-specific alteration of traces in central regions. Still, our findings showing a difference in the F8 component in particularly highlighted suggest this is a general feature of FND and a potential trait signature of FND pathophysiology. Yet future studies need to further investigate the local specificity and the differences to other disorders that show an attenuation of HEP in these frontal areas as well (e.g. in patients with atrial fibrillation or schizophrenia).^81,82^

### Cardiac differences

While the cardiac differences in terms of increased HR and decreased HRV in patients with FND compared with controls do not come surprising,^34,64,65^ they are relevant for HEP analysis, which is time-locked to the R-peaks. Also, as the timing of the identified interaction of group and condition was around 300 ms following the R-peak, which overlaps with previously discussed arousal-related HEP modulation.^32^ It also represents the temporal window where the diastole occurs, which is known as a phase that favours perception over action.^71^ Although autonomic differences such as elevated HR and reduced HRV have been implicated in neural alterations in other studies,^75^ we did not observe such correlations here, and the control for those cardiac difference would not diminish the group difference. Nonetheless, an increase in sympathetic tone is present in FND, and its potential involvement in interoceptive processing, beyond the measurements of this study, is further discussed in Supplementary material.

### Limitations

This study has several limitations that warrant consideration. Most notably, the higher resting HR in the FND group may have introduced variability in the R–R interval, increasing the likelihood of overlap between successive R-peaks and the 250–595 ms analysis window. Such overlap could affect late EEG components and potentially confound interpretation of interoceptive processing. However, excluding participants with shorter R–R intervals would disproportionately impact the FND group and risk sampling bias by removing clinically relevant cases, given that enhanced physical arousal is associated with the disorder. Therefore, we retained all individuals and accounted for group differences via statistical control. Nevertheless, residual confounding cannot be fully excluded. Another limitation is the lack of subtype-specific analyses (e.g. separating motor, from dissociative seizure subtypes) based on unequal distribution of symptoms. Additionally, while examiner-rated symptom severity was included, patient-reported disability scores are missing and restrict the assessment of the relationship between neural markers and symptom burden. Also, identified markers for interoceptive dysfunction need further investigation across different paradigms and in comparison to other clinical populations where interoception is involved, in order to more clearly define its relationship to FND. Further, the small time duration of >10 ms should be considered when interpreting our results, which derived from the explorative nature of our study. Finally, sample size was calculated based on a power calculation for a broader study including biological markers for interoception^37^ but not formalized based on HCT or HEP that involved a mixed FND populations.

## Conclusion

This study provides converging subjective, attentional and neurophysiological evidence for altered interoceptive processing in FND. While behavioural interoceptive accuracy (HCT) was preserved, self-reported interoceptive awareness was reduced and EEG analyses revealed attenuated late HEPs (F8; 250–595 ms) in FND. Attentional modulation of interoception was impaired, with diminished prefrontal recruitment and altered topographical dynamics during interoceptive focus. These findings highlight a disruption in the integration of bodily signals and related attentional control as key processes in interoceptive processing, identifying candidate neurophysiological markers for FND. Targeting interoceptive regulation in addition to attentional training may offer novel avenues for mechanistically informed treatment.

## Supplementary Material

fcaf503_Supplementary_Data

## Data Availability

Data for participants who consented further use of their data, along with the R-scripts used for obtaining the reported results are publicly available on GitHub.
