# Supplementary material for: Attenuated heartbeat-evoked potentials in functional neurological disorder
Source: Brain Commun. 2026 Jan 3;8(1):fcaf503. doi: 10.1093/braincomms/fcaf503 (PMC12810052; doi:10.1093/braincomms/fcaf503)
Supplement: fcaf503_Supplementary_Data [file fcaf503_supplementary_data.docx]

Supplementary Material:

Attenuated Heartbeat-evoked Potentials in Functional Neurological Disorder

Natascha Stoffel^1,2^, Michaël Mouthon^1^, Hang Yang^3^, Laure von der Weid^1^, Cristina Concetti^1^, Olaf Blanke^3,4^, Selma Aybek^1^

**Detailed description of assessing HEP:**

All participants were instructed to guide attention towards one of two signals, while keeping their eyes on a central fixation item. Participants were asked to focus their attention either on an interoceptive signal (heartbeat task was indicated by the symbol of a heart on the screen) or an exteroceptive-auditory signal (auditory task was indicated by white noise; symbol of headphones on the screen) while HEPs were recorded. **Supplementary** **Figure 1**. White noise was displayed via cable-bound noise-cancelling in-ear headphones during the entire duration of all trials, independent of where the focus of attention was directed. Each trial lasted for 20sec. Each condition was repeated ten times in a pseudo-randomized order, which was kept constant across all participants (for H=heart and S=sound; H-S-H-S-S-H-H-S-H-S-H-S-S-H-S-H-H-S-H-S). After each trial, participants were asked an associative question, e.g. “How much did the sound of white noise remind you of running water?” or “How much would you associated your heart perception in the last block with the color red?”) that they answered to as quickly as possible, within a maximum of 9 seconds, using a numeric scale from 1-10 on the screen. The answers to these questions were not analyzed as their sole purpose was to maintain vigilance and entertainment of the participant during the entire task. After an inter-trial interval (ITI) of 5-15s, the next trial would start, asking them again to focus on either the heartbeat or the white noise. Participants first completed a practice round for both conditions, where they could set the volume of the sound according to their comfort. After finishing the entire task, they answered three questions on a visual scale about each condition, indicating how well they could focus, how intensely they perceived the sound/their heartbeat and how difficult the task was for them (**Supplementary** **Table 4**). In total, the task lasted about 20 minutes.


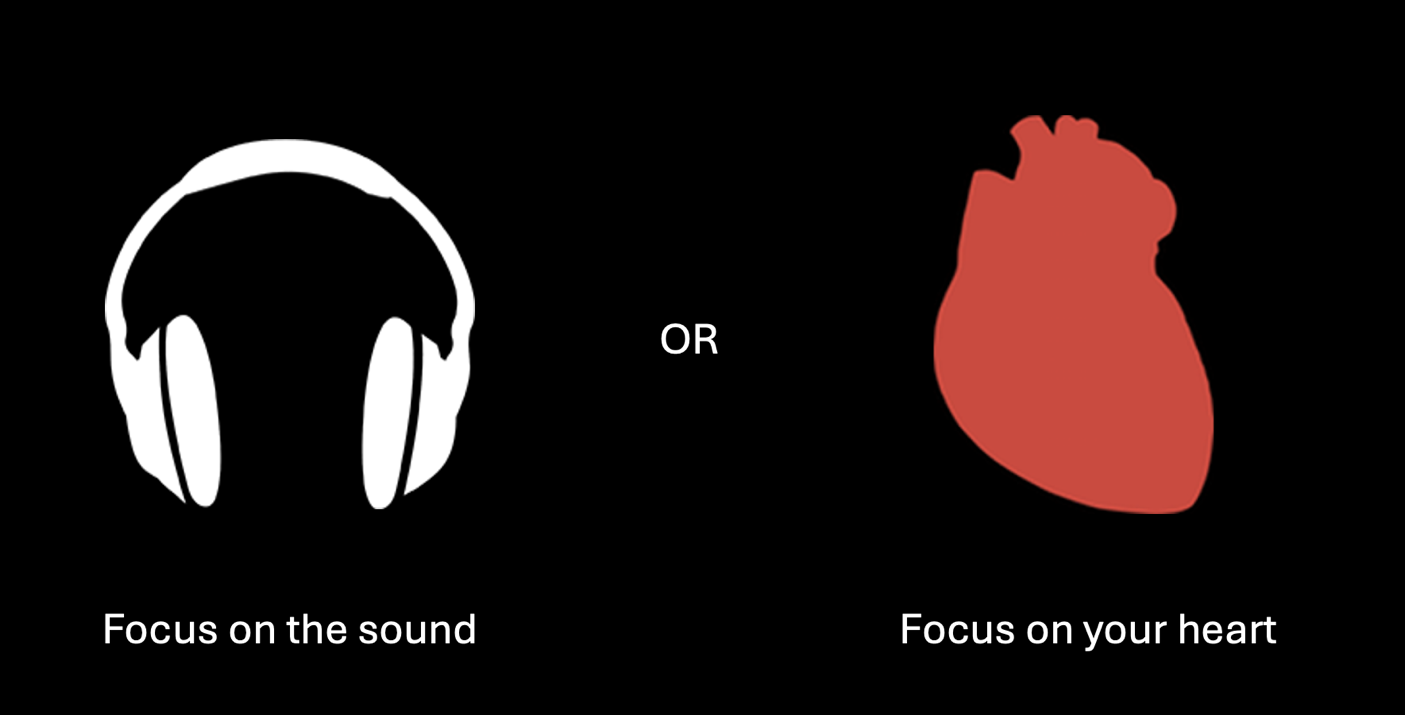
***Supplementary Figure 1 : Illustration of symbols on screen during the attention task.*** *Participants were instructed to fixate the symbol and focus on the sound or the heart, depending on which condition they were in. The symbol would be present for the entire focus-duration of 20seconds.*

| **Supplementary Table 1. Detailed Demonstration of Clinical Characteristics** | | | | |
| --- | --- | --- | --- | --- |
| **Variable** | **Overall,  N = 88** | **HC,  N = 48** | **FND,  N = 40** | **p-value** |
| Intake of Pain Medication | 15 (17%) | 3 (6.4%) | 12 (30%) | 0.005 |
| Intake of Psychotropic Medication | 19 (22%) | 2 (4.3%) | 17 (43%) | <0.001 |
| Intake of Sleep Medication | 8 (9.1%) | 0 (0%) | 8 (20%) | 0.001 |
| Intake of Other Medication | 20 (23%) | 7 (15%) | 13 (33%) | 0.072 |
| Comorbidity of ADS or AD(H)D | 5 (5.7%) | 1 (2.1%) | 4 (10%) | 0.2 |
| Comorbidity of Psychiatric Disorder | 14 (16%) | 3 (6.3%) | 11 (28%) | 0.009 |
| Comorbidity of Hormonal Dysfunction | 3 (3.4%) | 1 (2.1%) | 2 (5.0%) | 0.6 |
| Comorbidity of other Diagnosis | 12 (14%) | 3 (6.3%) | 9 (23%) | 0.033 |
| FEMALE only: Intake of Hormonal Contraception | 12 (18%) | 3 (8.6) | 9 (30) | 0.051 |
| FEMALE only: in Menopause | 11 (17%) | 7 (20%) | 4 (13%) | 0.5 |
| FEMALE only: natural, regular menstrual cycle | 35 (88%) | 21 (84) | 14 (93%) | 0.6 |
| *Note: Numbers represent count of existing clinical characteristics (one participant can also have more than one characteristics), and in brackets percentage from the population. P-vales show differences between groups using Fisher’s exact test.* | | | | |

| **Supplementary Table 2. Subtypes present in the FND cohort** | | | |
| --- | --- | --- | --- |
| **Variable** | **Overall,  N = 40** | **female,  N = 30** | **male,  N = 10** |
| **Symptoms** |  |  |  |
| Functional Dissociative Seizures | 7 (17.5) | 7 (23.3) | 0 (0.0) |
| motor + symptoms | 17 (42.5) | 12 (40.0) | 5 (50.0) |
| motor - symptoms | 27 (67.5) | 19 (63.3) | 8 (80.0) |
| sensory symptoms | 20 (50.0) | 16 (53.3) | 4 (40.0) |
| dizziness (PPPD) | 2 (5.0) | 2 (6.7) | 0 (0.0) |
| cognitive symptoms | 4 (10.0) | 4 (13.3) | 0 (0.0) |
| **Diagnosis** |  |  |  |
| 44.5 Functional Dissociative Seizures | 3 (7.5) | 3 (10) | 0 (0) |
| 44.4 motor FND | 16 (40.0) | 10 (33.3) | 6 (60.0) |
| 44.6 sensory FND | 2 (5.0) | 2 (6.7) | 0 (0) |
| 44.7 mixed FND | 19 (47.5) | 15 (50) | 4 (40) |
| *Note: For “Symptoms”, the numbers represent counts (and percentage) of existing symptoms, meaning that one patient can also have more than one symptom subtype. For “Diagnosis” each patient has only one diagnosis.* | | | |

Patients in the FND group on average had symptoms for 76 months (ranging from 12-305 months).

**Scholarity**

Scholarity levels per group are indicated in **Supplementary** **Table 3,** representing the highest educational degree obtained by participants. Fisher’s exact test for count data indicated that the distribution of highest education levels differed between HC and FND, *p* = 0.049.

| **Supplementary Table 3: Scholarity per group** | | |
| --- | --- | --- |
| **Scholarity, Count (%)** | **HC, N = 48** | **FND, N = 40** |
| none | 0 (0) | 1 (2.6) |
| compulsory school (primary and secondary school; 9 years) | 3 (7.3) | 6 (15.8) |
| apprenticeship | 10 (24.4) | 19 (50.0) |
| Matura (cantonal highschool) | 6 (14.6) | 6 (15.8) |
| Higher technical collage (HF or FH) | 13 (31.7) | 8 (21.1) |
| bachelor's degree | 4 (9.8) | 2 (5.3) |
| master's degree | 12 (29.3) | 3 (7.9) |
| PhD | 0 (0.0) | 1 (2.6) |
| other | 1 (2.4) | 0 (0.0) |


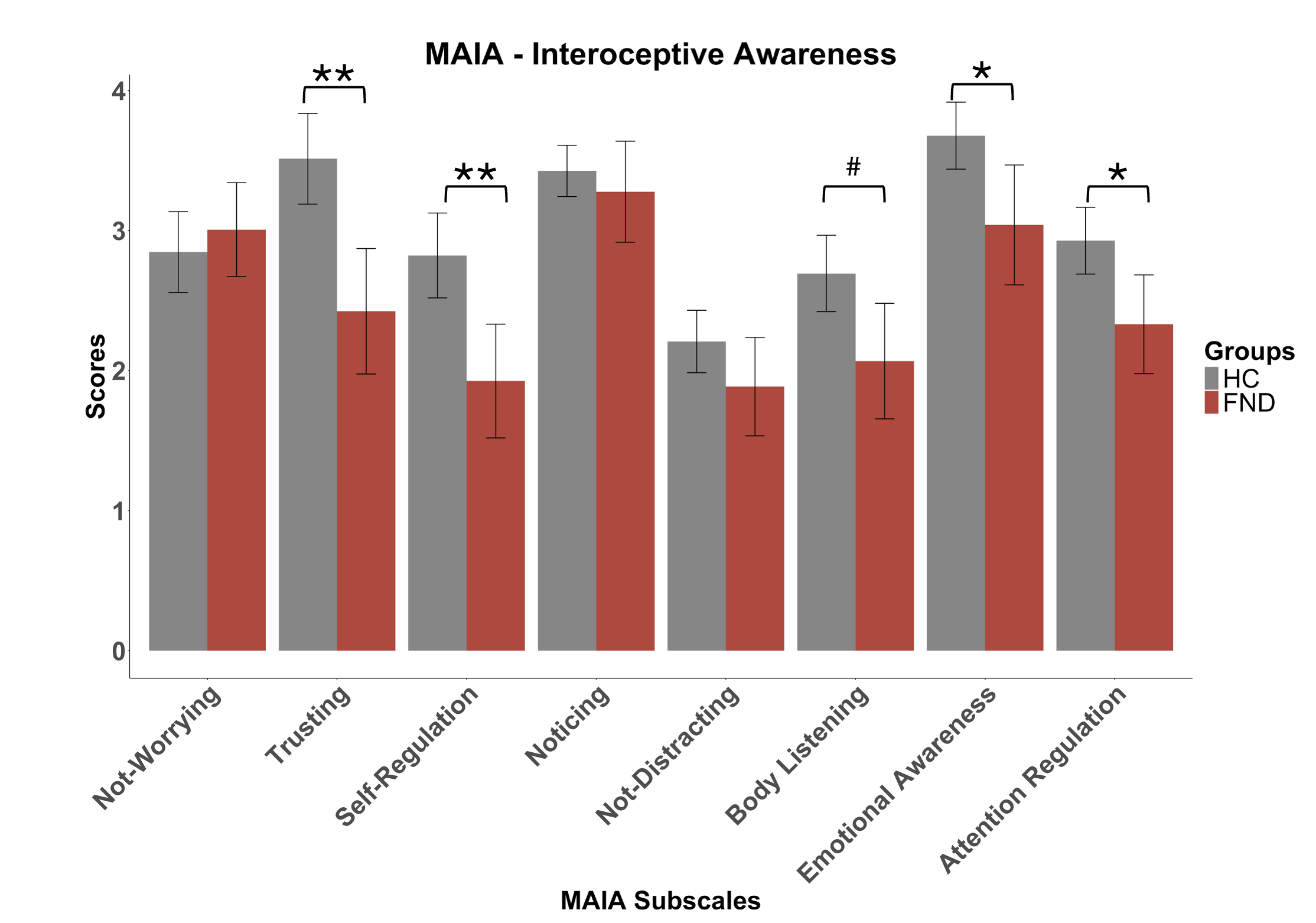


***Supplementary Figure 2 : Subscales of Multidimensional Assessment of Interoceptive Awareness (MAIA)*** *Bars represent group mean scores of each subscale in the MAIA questionnaire, separated for groups (N = 48 Healthy Controls; HC and N = 40 patients with Functional Neurological Disorder; FND), showing 95% confidence intervals. Statistical comparisons were conducted using t-tests per subscale with p-values adjusted for multiple comparisons using False Discovery Rate.Levels of significance: # p = 0.05, ** *p < 0.05, ** p < 0.01*

| **Supplementary Table 4. Detailed Ratings on the Heartbeat Counting Task (HCT)** | | | | |
| --- | --- | --- | --- | --- |
| **Variable** | **Overall,  N = 88** | **HC,  N = 48** | **FND,  N = 40** | **p-value** |
| HCT mean accuracy | 0.5 (0.5) | 0.5 (0.5) | 0.4 (0.4) | 0.060 |
| HCT mean confidence | 20.6 (36.5) | 21.7 (34.7) | 19.8 (33.9) | 0.4 |
| HCT mean intensity | 25.0 (22.8) | 25.0 (26.3) | 24.2 (23.1) | 0.4 |
| HCT mean easiness | 23.8 (26.7) | 25.7 (25.0) | 20.7 (28.0) | 0.3 |
| HCT change in accuracy (neutral to arousal) | 0.0 (0.3) | 0.0 (0.2) | 0.0 (0.2) | 0.7 |
| HCT change in confidence (neutral to arousal) | 0.0 (26.3) | 3.2 (28.3) | 0.0 (23.8) | 0.5 |
| Interoceptive Awareness: MAIA | 23.1 (7.5) | 24.8 (4.8) | 19.9 (8.2) | 0.001 |
| Interoceptive Accuracy Self-Report: IAS | 85.0 (19.0) | 89.0 (14.3) | 79.5 (27.0) | 0.020 |
| *Note: Numbers report median scores of variables assessed during the Heartbeat Tracking Task (HCT)  with IQR in brackets. P-values show group differences using Wilcoxon rank sum test* | | | | |

| **Supplementary Table 5: Rating in Attention Task** | | | | |
| --- | --- | --- | --- | --- |
| **Variable** | **Overall,**  **N = 88** | **HC,**  **N = 48** | **FND,**  **N = 40** | **p-value** |
| Easiness to perceive Heartbeat | 16.0 (28.3) | 26.5 (27.3) | 12.0 (23.0) | 0.002 |
| Easiness to perceive Sound | 81.5 (26.5) | 88.0 (25.5) | 78.5 (49.5) | 0.2 |
| Ability to focus on Heartbeat | 30.5 (37.8) | 33.5 (33.3) | 28.0 (43.5) | 0.3 |
| Ability to focus on Sound | 82.5 (21.3) | 84.0 (19.5) | 81.5 (21.5) | 0.3 |
| Intensity of perceived Heartbeat | 19.0 (26.5) | 23.5 (29.3) | 18.5 (26.0) | 0.15 |
| Intensity of perceived Sound | 87.0 (23.8) | 87.0 (18.0) | 86.0 (28.5) | 0.9 |
| *Note: Table displaying median and (IQR) for group differences in the rating of the attention task, with p-value derived from Wilcoxon rank sum test* | | | | |

## **Targeted local (control) analysis**

### To control further the specificity of the electrode sites, and run a similar control analysis as Flasbeck et al., (2024) we conducted four times 2x3x2 ANOVA (group x site x condition), separately for four defined regions. Please note the difference in this analysis nonetheless: instead of within-group difference (during vs before seizure), we have a between-group difference (HC vs FND), and instead of latency (early vs late time window), we have a condition effect (interoceptive vs exteroceptive focus). However, the chosen regions to test, are the same. The first region was our targeted electrodes (F7, Fz and F8), while there were three control regions, namely frontopolar Region 1 (Fp1, Fpz, Fp2), mid-frontal Region 2 (C3, Cz, C4), as well as central Region 3 (F3, Fz and F4).

Normality testing (Shapiro–Wilk) indicated non-normal distributions of EEG amplitudes in all regions (p < 0.05), whereas Levene’s tests confirmed homogeneity of variance across groups and conditions (p > 0.05). Therefore, we report both the classical ANOVA, as well as the results from the more robust non-parametric ART (i.e., aligned rank transform) analysis per region.

First, the mixed-design ANOVA including site as a factor for our target electrodes (F7, Fz and F8) revealed a main effect of **group** (F(1, 86) = 5.39, p = 0.023), but no interactions with group. The ART model replicated these finding with a group main effect (F(1, 86) = 5.50, p = 0.021). Thus, EEG amplitudes in the target region were generally reduced in the FND group relative to HCs, however this was consistent across electrode-site and conditions within this region.

*Frontopolar Control Region 1 (Fp1, Fpz, Fp2):* Our ANOVA as well as the ART analysis indicated no group or condition effect or interaction, suggesting that only EEG amplitudes varied by electrode site within the region, with no influence of group or condition.

*Mid-frontal* *Control Region 2 (F3, Fz, F4)*: While the classical ANOVA showed no main effect of group (F(1, 86) = 3.05, p = 0.084), the non-parametric approach using ART revealed a main effects of group (F(1, 86) = 6.14, p = 0.015), without interacation effects. These results point towards lower EEG amplitudes in the FND group across the mid-frontal electrodes, which is independent of condition.

*Central Control Region 3 (C3, Cz, C4):* Here, the ANOVA revealed a significant interaction between group and condition (F(1, 86) = 4.20, p = 0.043), with post-hoc contrasts (estimated marginal means) indicating, that this interaction was driven by a condition effect within the FND group (t(86) = –2.65, p = 0.010). Lower amplitudes were found during the interoceptive condition of focusing on the heart, compared to focusing on the sound (exteroceptive control condition), indicating that guiding the attention toward the heart attenuates the central traces in this time-period overall. However, using the non-parametric robus ART analysis, this interaction is only a trend (F(1, 86) = 3.63, p = 0.060).

In conclusion, this control analysis further supports further the group effect of the chosen eletrodes (F7, Fz, F8), while there is evidence that this group difference extends towards more central region, and that there is a FND-specific interaction with the attention condition in central regions.

##
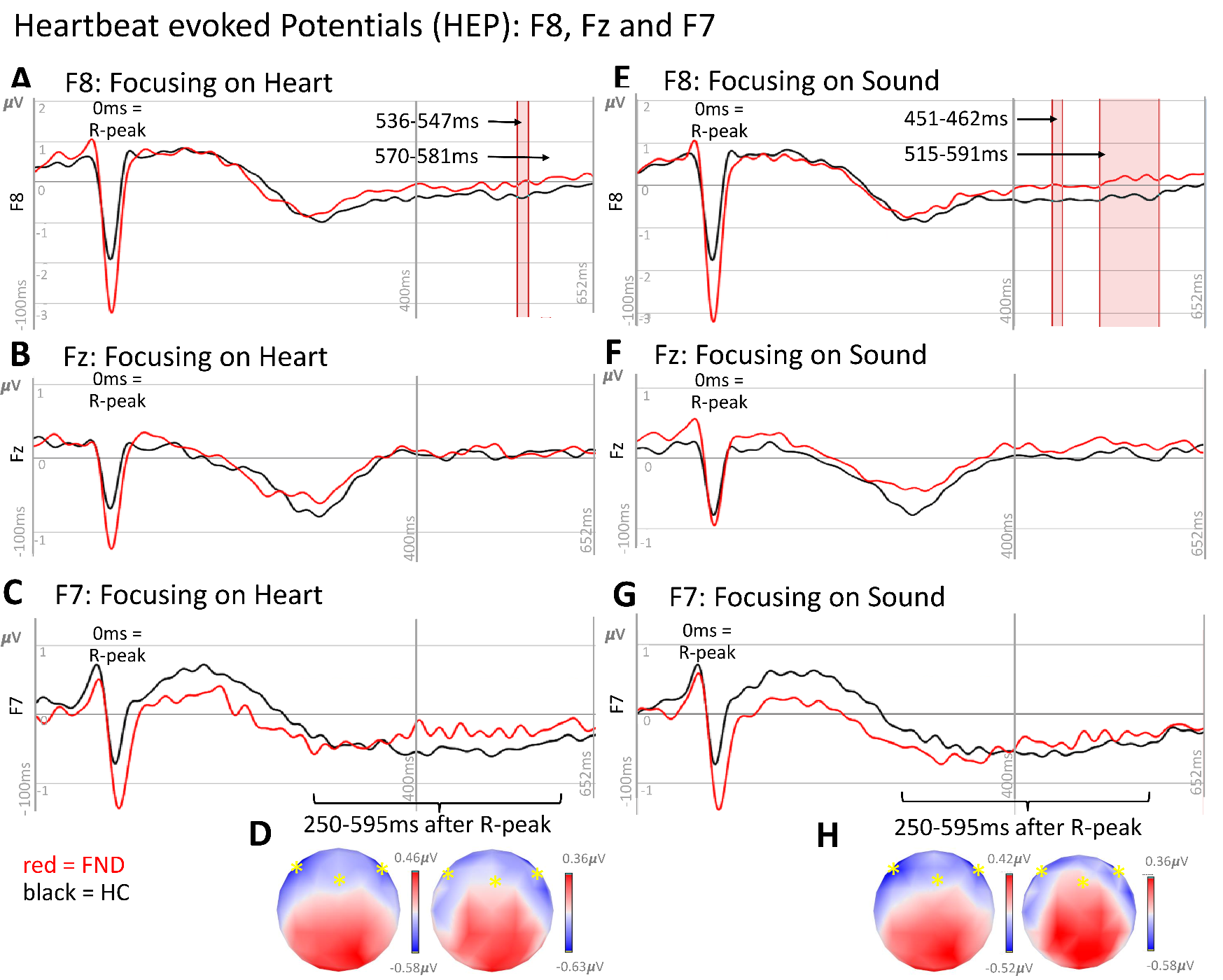
**Targeted analysis (F8, Fz, F7) – full data**

***Supplementary Figure 3: Illustration of all three traces from the targeted analysis per group and condition.*** *Grand average for electrode F8, Fz and F7, associated with the Heartbeat evoked potential (HEP) for N = 40 Functional Neurological Disorder; FND in red traces and N = 48 healthy controls; HC in black traces.* ***A-C)*** *shows the interoceptive condition of focusing on the heart, separate for a each electrode position (F8, Fz and F7) and* ***E-G)*** *shows the control condition of focusing on the sound, separate for each electrode position (F8, Fz, F7). The red highlighted area in A and E represent the time interval where F8 became significant in the group comparison separately per condition (between group randomization analysis in Cartool; p < 0.05, after FDR correction for multiple comparison. No sig difference for any time interval identified for Fz as visible in B and F or F7 (as visible in C and G).* ***D)*** *descriptively shows the topography for the full duration of the tested interval, i.e. 250-595ms, with the yellow asterix pointing out the position of the three selected electrodes F8 (right side), Fz (central) and F7 (left side) in the interoceptive condition and* ***H*** *descriptively shows the topography in the control condition respectively.*

## **Explorative local trace analysis for interoceptive condition: F1**

## Performing a randomization test between groups in the interoceptive condition only an early effect of F1 was detected at 185-210ms following R-peak (**Supplementary Figure 4**). This group difference remained when running a linear regression with the extracted signal controlling for covariates of sex, age (F(3, 84) = 5.10, p = 0.0027), for cardiac difference including either HR and HRV (F(3, 84) = 4.59, p = 0.0051), or the ECG ampltide F(2, 85) = 8.53, p = 0.0004)), as well as controlling for intake of medication and affective symptoms (*F*(3, 84) = 6.13, *p* = 0.0008). The repeated measures ANOVA examining the interaction at this time interval between group and condition identified, next to the identified main effect of group (F(1,86) = 9.43, p = 0.003), weak evidence for an interaction approaching significance threshold (F(1,86) = 3.80, p = 0.054). No other traces were identified exploratively over the full scalp for the interoceptive condition.


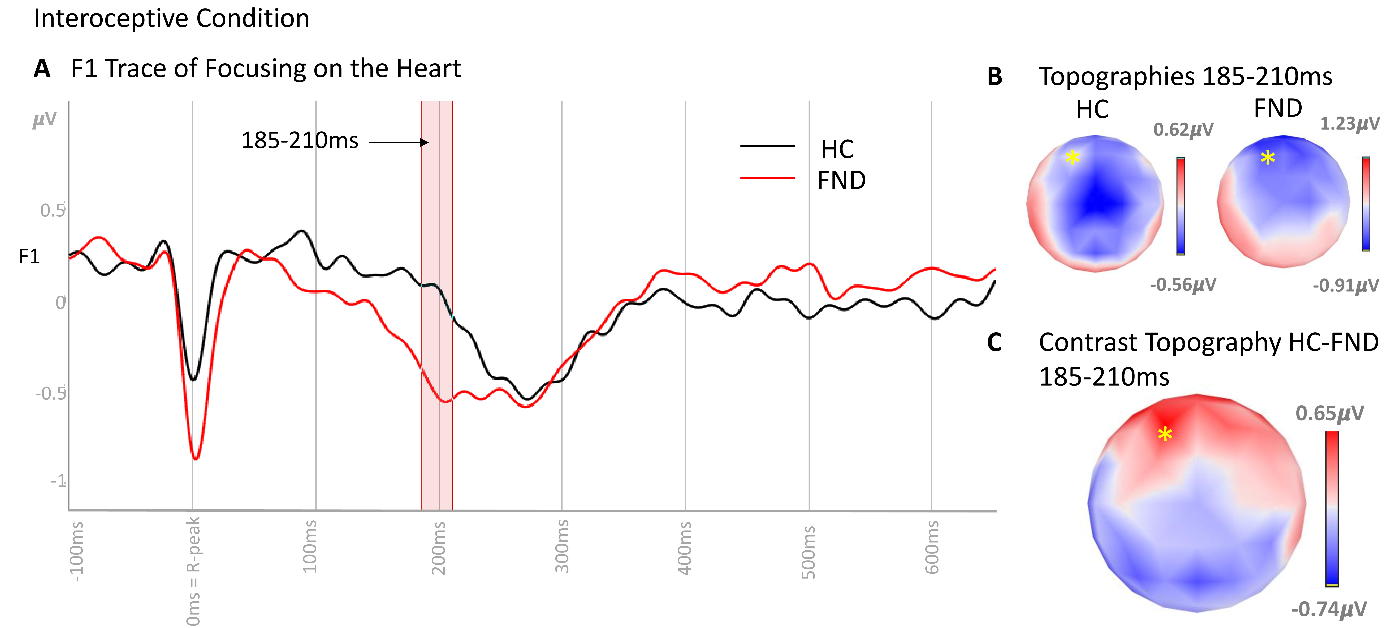


***Supplementary Figure 4: Heartbeat-evoked potentials (HEPs) at electrode F1 for both groups* during *interoceptive condition of focusing on the heartbeat. A)*** *Shows the trace of* *mean average of N = 40 patients with Functional Neurological Disorder; FND in red trace, and N = 48 healthy controls; HC in black trace, portraying the time period significant for the between-group randomization test at the level of p < 0.05 after correcting for multiple comparison at 185-210ms after R-peak highlighted. No baseline correction was implemented.* ***B)*** *Shows the corresponding topographies per group, along with* ***C)*** *the difference of topography below (grand average of HC – grand average of FND) illustrating an overall enhanced frontal negativity of patients compared to controls in this early time interval.*

Exploratory trace analysis revealed an enhanced early frontal negativity (185–210ms at F1) in patients with FND during interoceptive attention. This early HEP component likely reflects processing of cardiac signalling rather than higher-order cognitive processes typical of later HEPs.^1^ Its latency and topography resemble the N2 component, associated with novelty detection and salience processing in analysis of event-related potentials,^2^ yet cannot be interpreted as easily as an N2 from being locked to the R-peak instead of an external stimulus. Attenuated auditory responses to heartbeat-related signals have been linked to interoceptive predictive coding and bodily self-consciousness before,^3^ the HEP itself is thought to index salience-weighted interoceptive prediction errors^4^ and reduced interoceptive monitoring has been linked to altered self-processing.^5^ Taken together, this novel finding of the early, increased frontal response in FND may reflect heightened novelty of cardiac signals due to reduced predictability or attenuation but this interpretation considers caution. This effect persisted after controlling for HR and HRV, arguing against a purely physiological explanation, and correlated with IAS scores, suggesting modulation by interoceptive trait accuracy.

## **Explorative local trace analysis for exteroceptive condition: F8 and P6**

Performing a randomization test between groups in the exteroceptive condition further aimed to identify any electrodes at any time interval that is different between the two groups particularly for the control condition. At electrode F8, a group difference was identified for the control condition at 540-550ms post-R-peak. This group difference remained significant after controlling for sex, age and the cardiac covariates (HRV and heart rate), *b* = 0.45, *p* = 0.003, *F*(5,81) = 3.20, *R²* = 9.84%, *p* = 0.011). Finally, also when including intake of psychotropic medication and affective symptoms in addition to sex and age as covariates of no-interest, the group effect remained (*b* = 0.47, *p* = 0.007, *F*(5,80) = 2.76, *R²* = 13.77%, *p* = 0.024), suggesting a robust effect of group at this frontal site.

A similar group effect was observed at P6, where FND patients showed a decreased amplitude 620-630ms post-R-peak. Further control analysis supported the group difference even when controlling for sex and age and cardiac differences (*b* = -0.37, *p* = 0.005, *F*(5,81) = 3.20, *R²* = 11.33%, *p* = 0.011). However, with adding the affective symptoms and adjusting for medication intake, along with sex and age, the group effect would disappear (*b* = -0.26, *p* = 0.074, *F*(5,80) = 4.15, *R²* = 20.58%, *p* = 0.0021). These results indicate that the group differences at P6 are somewhat sensitive to covariate inclusion of affective symptoms and medication but remain of medium effect size.

**Differences in Condition: Topographical Differences**

The tANOVA analysis identified a main effect for the factor of condition, with a topographical difference at 510-524ms after R-peak (p = 0.005). This identified time interval lies within the same time frame where Petzschner *et al.* (2019) found a difference in GFP between condition.^6^ Using sLORETA to visualize the source of this topographical difference, we identify BA 18, the cuneus and the occipital lobe as the origin (t = 3.03, *p* > 0.05), which is consistent with running the same analysis separately for controls (*t* = 2.54, *p* > 0.05). For patients with FND only, it was a deactivation of the BA 6, the precentral gyrus and the frontal lobe that was specific to the focusing of the heart (*t* = -3.01, *p* > 0.05), thus the effect of condition between group and the replication of Petzschner *et al,*’s interval of interest was mainly driven by controls. Also note that in the same time interval there is also a main effect of group for the tTANOVA analysis (p = 0.020).

We extracted the GMD score 510-524ms post R-peak via EEGpal to test for further correlations with other variables. A negative correlation of the differential topographical activation for the focus on the heart would be correlated with interoceptive accuracy (using the HCT: *r = -0.25, p = 0.034*) after FDR correction for multiple comparison. Further, the GMD at 510m post R-peak when focusing on the heart was also correlated with self-rated somatoform dissociation scores (r = 0.35, p < 0.001). This association remained when running a linear regression for FND group only (F(5,34) = 6.89, R^2^=43%, p < 0.001) controlling for sex, age, affective symptoms (t = 3.68, p=8.09e-04), while medication was also associated with SDQ-20 (t = 2.05, p= 0.048). **Supplementary** **Figure 5.**


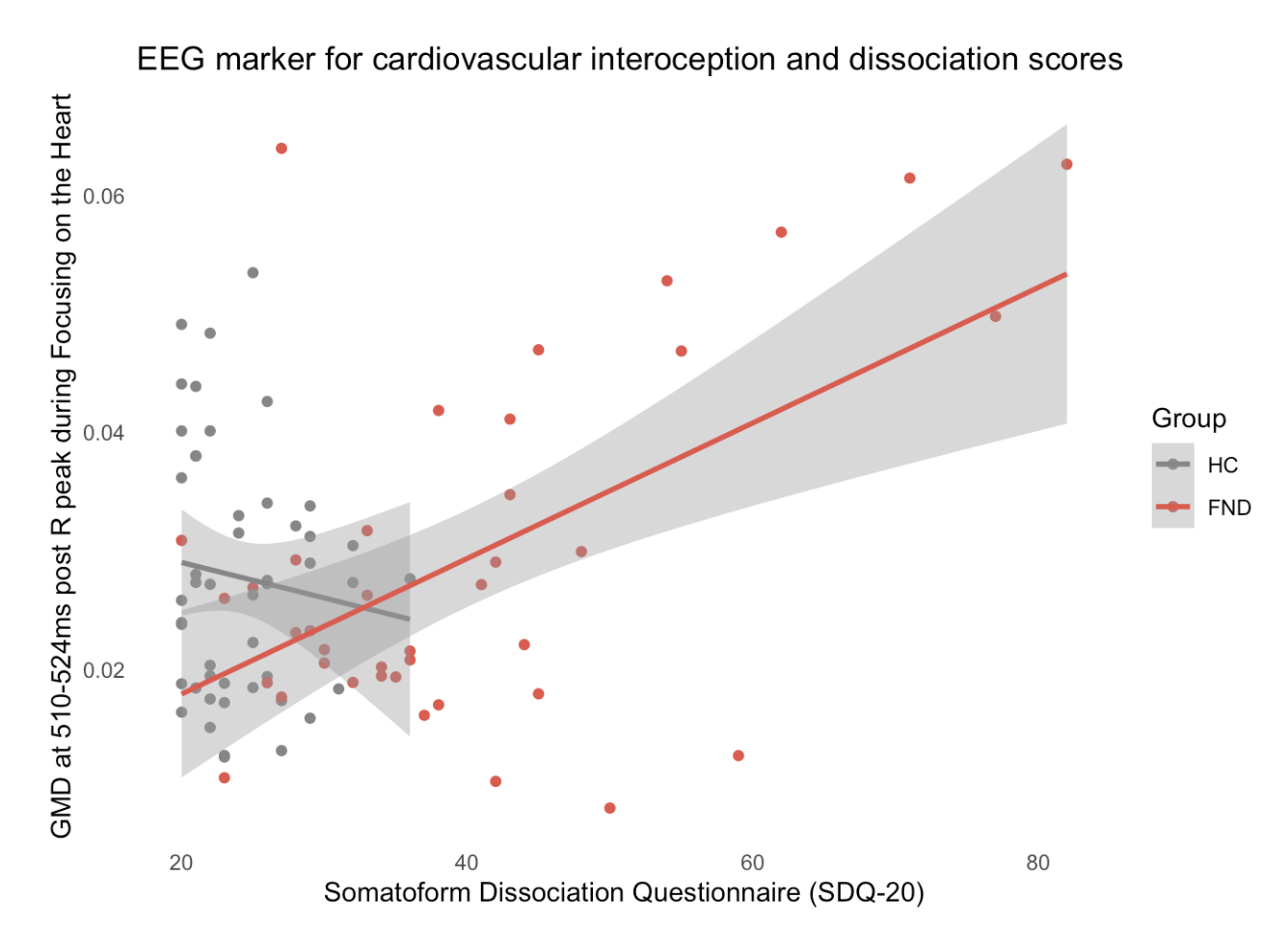
 ***Supplementary Figure 5: Somatoform Dissociation correlated with Global Map Dissimilarity (GMD) at 510ms post R-peak uniquely for patients with Functional Neurological Disorder (FND).*** *For higher scores on the Somatoform Dissociation Questionaire (SDQ-20), N = 40 FND patients also showed a higher GMD at 510-524ms after R-peak, compared to N = 48 healthy control group (HC). This time interval has been detected to be significantly different between condition, (independent of group), identified in the multifactorial global analysis using randomization statistics in RAGU. The SDQ-20 and GMD association was calculated with Pearson correlation, leading to r = 0.35, p < 0.001 in the FND group. This association for FND patients remains when controlling for sex, age, affective symptoms and the intake of medication (t = 3.68, p<0.001). Each participants represents as datapoint in the jitterplot.*

This main effect of condition lies within the same time interval as the GFP identified as an effect of condition in the original article of Petzschner *et al.,* (2019) with a healthy male cohort^6^. We may not have been able to directly replicate their findings due to the heterogeneity of our sample, which included both controls and patients with FND. Additionally, our study had mostly female participants, whereas their original study included only nineteen males, and electrophysiological processing differences between sexes may have influenced the results.^7^

For our topographical difference in the same time interval, we identified the cuneaus and the BA 18; the visual association area, being uniquely activated for the focus on the heartbeat compared to the sound. This effect was predominantly driven by the control group. Previous findings in a healthy population show a correlation of interoceptive attention, an increased HEP and specifically a decrease in parieto-occipital alpha-band activation.^8^ As alpha band activations are interpreted to represent inhibitory mechanisms facilitating a specific recruiting of task-relevant regions only,^9^ our identified source localization as an effect of condition might represent the same mechanisms. Arguably, we can interpret our findings of GMD for the effect of condition in these lights, especially as we find a topographical activation for the interoceptive focus on the occipital regions as well, mainly apparent in the healthy control group. Notably, this GMD at 510ms post-R peak was further negatively correlated with HCT accuracy across groups, and negatively with somatoform dissociation in FND. Finally, in patients with FND, the GMD at 510ms post-R peak during heart-focused attention (reflecting the inability to engage the occipital-cuneus network for interoceptive attention) was linked to self-reported somatoform dissociation symptoms. This association remained robust after controlling for sex, age, affective symptoms, and medication. Thus, the reduced activation of the cuneus and occipital regions during interoceptive compared to exteroceptive attention emerges as both an electrophysiological marker of interoceptive performance, as well as a robust clinical indicator of somatoform dissociation in FND.

**Cardiac regulation and interoceptive dysfunction**

We are here trying to discuss the heightened sympathetic activity (such as higher HR and lower HRV) not only as confounding variables of the presented results in the manuscript so far but further integrate it more in the understanding of underlying pathophysiology, that goes beyond what we were able to measure and control for in this presented study. Nonethless, important to note is that the cardiac deacceleration has not only been associated with parasympathetic activity, but been considered as a dynamic process that minimizes exteroceptive processing, when action is not needed. HR typically slows during heartbeat-focused tasks, indicating a physiological mechanism facilitating interoceptive attention.^10^ Which is also why we tested for potential within-group differences in cardiac modulation, separate per group. Using pairwise tests (t-test for normally distributed HR, and Wilcoxon-test for non-normally distributed HRV) we identified only a within-group difference for HR for FND (p = 0.003), to show a slowing of HR during the interoceptive condition. In HCs, this expected effect was not present, but arguably because they had a lower HR overall, or because they would already deaccelerate the heartrate for any focusing task (whether it is exteroceptive sound, or interoceptive heartbeat signals). This may suggest that patients with FND experience greater autonomic activation at baseline and must exert more regulatory effort to engage interoceptive attention. Previous work has shown that elevated arousal is associated with reduced HEP in functional seizures, though this relationship vanishes during acute episodes, suggesting a disruption in arousal–interoception integration.^11^ Also, in the functional/dissociative seizure subtype, cardiac modulation was impaired (i.e. less alteration in perceived intensity for presented stimuli depending on being in the systolic compared to diastolic phase) and was associated with higher interoceptive accuracy task and lower symptom severity.^12^ Importantly, the differences in overall sympathetic tone do not explain difference in HEPs reported in our study, suggests no direct association to the overall HR and HRV. Yet, knowing that an increase in HR, is associated with an attenuation of cortical excitability and thus an attenuation of perception,^13^ our results with group differences both in the diastolic phase and overall increased HR and lower HRV open future study approaches for including or targeting autonomic regulation more specifically. In such, systolic compared to diastolic phases could be a potential site of involvement, given its role in modulating interoceptive integration and bodily awareness.

**Supplementary References**

1. Park S, Ha J, Kim L. Anti-Heartbeat-Evoked Potentials Performance in Event-Related Potentials-Based Mental Workload Assessment. *Front Physiol*. 2021;12. doi:10.3389/fphys.2021.744071

2. Bocquillon P, Bourriez JL, Palmero-Soler E, Molaee-Ardekani B, Derambure P, Dujardin K. The spatiotemporal dynamics of early attention processes: A high-resolution electroencephalographic study of N2 subcomponent sources. *Neuroscience*. 2014;271:9-22. doi:10.1016/j.neuroscience.2014.04.014

3. van Elk M, Lenggenhager B, Heydrich L, Blanke O. Suppression of the auditory N1-component for heartbeat-related sounds reflects interoceptive predictive coding. *Biological Psychology*. 2014;99:172-182. doi:10.1016/j.biopsycho.2014.03.004

4. Engelen T, Solcà M, Tallon-Baudry C. Interoceptive rhythms in the brain. *Nat Neurosci*. 2023;26(10):1670-1684. doi:10.1038/s41593-023-01425-1

5. Babo-Rebelo M, Wolpert N, Adam C, Hasboun D, Tallon-Baudry C. Is the cardiac monitoring function related to the self in both the default network and right anterior insula? *Philosophical Transactions of the Royal Society B: Biological Sciences*. 2016;371(1708):20160004. doi:10.1098/rstb.2016.0004

6. Petzschner FH, Weber LA, Wellstein KV, Paolini G, Do CT, Stephan KE. Focus of attention modulates the heartbeat evoked potential. *NeuroImage*. 2019;186:595-606. doi:10.1016/j.neuroimage.2018.11.037

7. Ramos-Loyo J, González-Garrido AA, Llamas-Alonso LA, Sequeira H. Sex differences in cognitive processing: An integrative review of electrophysiological findings. *Biological Psychology*. 2022;172:108370. doi:10.1016/j.biopsycho.2022.108370

8. Kritzman L, Eidelman-Rothman M, Keil A, Freche D, Sheppes G, Levit-Binnun N. Steady-state visual evoked potentials differentiate between internally and externally directed attention. *NeuroImage*. 2022;254:119133. doi:10.1016/j.neuroimage.2022.119133

9. Haegens S, Osipova D, Oostenveld R, Jensen O. Somatosensory working memory performance in humans depends on both engagement and disengagement of regions in a distributed network. *Human Brain Mapping*. 2010;31(1):26-35. doi:10.1002/hbm.20842

10. Candia-Rivera D, Sappia MS, Horschig JM, Colier WNJM, Valenza G. Confounding effects of heart rate, breathing rate, and frontal fNIRS on interoception. *Sci Rep*. 2022;12(1):20701. doi:10.1038/s41598-022-25119-z

11. Flasbeck V, Jungilligens J, Lemke I, et al. Heartbeat evoked potentials and autonomic arousal during dissociative seizures: insights from electrophysiology and neuroimaging. *BMJ Neurol Open*. 2024;6(1):e000665. doi:10.1136/bmjno-2024-000665

12. Koreki A, Garfinkel S, Critchley H, et al. Impaired cardiac modulation in patients with functional seizures: Results from a face intensity judgment task. *Epilepsia*. 2023;(64):3073-3081. doi:10.1111/epi.17761

13. Skora LI, Livermore JJA, Roelofs K. The functional role of cardiac activity in perception and action. *Neuroscience & Biobehavioral Reviews*. 2022;137:104655. doi:10.1016/j.neubiorev.2022.104655
